# Expression Map of the Human Exome in CD34+ Cells and Blood Cells: Increased Alternative Splicing in Cell Motility and Immune Response Genes

**DOI:** 10.1371/journal.pone.0008990

**Published:** 2010-02-01

**Authors:** Sylvie Tondeur, Céline Pangault, Tanguy Le Carrour, Yoann Lannay, Rima Benmahdi, Aurélie Cubizolle, Said Assou, Véronique Pantesco, Bernard Klein, Samir Hamamah, Jean-François Schved, Thierry Fest, John De Vos

**Affiliations:** 1 CHU Montpellier, Institute for Research in Biotherapy, Hôpital Saint-Eloi, Montpellier, France; 2 INSERM, U847, Montpellier, France; 3 Université MONTPELLIER1, UFR de médecine, Montpellier, France; 4 CHU Rennes, laboratoire d'Hématologie, Rennes, France; 5 INSERM U917, Université de Rennes 1, UFR de Médecine, Rennes, France; 6 CHU Montpellier, Unité biologie clinique d'AMP - DPI, Hôpital Arnaud de Villeneuve, Montpellier, France; 7 CHU Montpellier, Laboratoire d'Hématologie, Hôpital Saint-Eloi, Montpellier, France; National University of Ireland Galway, Ireland

## Abstract

**Background:**

Hematopoietic cells are endowed with very specific biological functions, including cell motility and immune response. These specific functions are dramatically altered during hematopoietic cell differentiation, whereby undifferentiated hematopoietic stem and progenitor cells (HSPC) residing in bone marrow differentiate into platelets, red blood cells and immune cells that exit into the blood stream and eventually move into lymphoid organs or inflamed tissues. The contribution of alternative splicing (AS) to these functions has long been minimized due to incomplete knowledge on AS events in hematopoietic cells.

**Principal Findings:**

Using Human Exon ST 1.0 microarrays, the entire exome expression profile of immature CD34+ HSPC and mature whole blood cells was mapped, compared to a collection of solid tissues and made freely available as an online exome expression atlas (Amazonia Exon! : http://amazonia.transcriptome.eu/exon.php). At a whole transcript level, HSPC strongly expressed EREG and the pluripotency marker DPPA4. Using a differential splicing index scheme (dsi), a list of 849 transcripts differentially expressed between hematopoietic cells and solid tissues was computed, that included NEDD9 and CD74. Some of these genes also underwent alternative splicing events during hematopoietic differentiation, such as INPP4B, PTPLA or COMMD6, with varied contribution of CD3+ T cells, CD19+ B cells, CD14+ or CD15+ myelomonocytic populations. Strikingly, these genes were significantly enriched for genes involved in cell motility, cell adhesion, response to wounding and immune processes.

**Conclusion:**

The relevance and the precision provided by this exon expression map highlights the contribution of alternative splicing to key feature of blood cells differentiation and function.

## Introduction

The normal function of cells depends on the accurate expression of a large array of protein-coding messenger RNA (mRNA). One important source of diversity in mRNAs takes place in the processing of the pre-mRNA that results in different splice variants. It is estimated that more than 75% multi exon genes undergo alternative splicing in one or more tissues [1], providing prodigious opportunities for enrichment of the transcriptome and the proteome from our finite genome. The functional importance of AS is even more highlighted by the finding that about 10% of genetic diseases caused by point mutation affect the spliceosome formation [2]. The importance of this level of gene regulation has also been recognized in the hematopoietic system, especially immune cells, but this knowledge is still very limited [3], [4]. Blood is a fluid tissue composed of different cell types which performs different functions in the body encompassing oxygenation, defense against infectious agents and hemostasis. Blood cells share nevertheless numerous functional properties that distinguish them from other solid tissues, including cell motility and, for white blood cells, immune functions. Of note, these functions mature during hematopoietic cell differentiation and become fully operative at the moment when hematopoietic cells leave bone marrow or other organs of the immune system towards the peripheral blood circulation. The understanding of hematopoietic cell functions has been largely established by the identification of a large number of effector genes expressed in hematopoietic cells, but has not been extensively substantiated at the AS level.

As the catalog of our coding exons, the “exome”, is improving in definition, we took the opportunity to analyze the expression of over 1 million known or predicted human exons in immature hematopoietic progenitor cells & hematopoietic stem cells (HSPC) and mature whole blood cells using the GeneChip Human Exon 1.0 ST (Affymetrix) microarray. Indeed, these microarray do not rely on a limited catalog of splicing specific probes, but instead cover a very large list of exons identified or predicted so far, thus limiting biases toward previous known AS [1], [5]. By analyzing their exome expression profile, we were able to establish the alternative splicing events that characterize hematopoietic cells and that mark hematopoietic differentiation. Interestingly, these genes were significantly enriched for genes involved in cell motility, cell adhesion, response to wounding and immune processes. We experimentally confirmed these computational results using qRT-PCR on purified blood cell populations. These results shed light on a level of gene expression whose role is known, but still rarely taken into account in functional studies due to the lack of access to this information. The creation of an exon expression atlas covering 13 types of tissues including hematopoietic cells (http://amazonia.transcriptome.eu/exon.php) provides a mean to disseminate this knowledge.

## Materials and Methods

### Sample Preparation and Microarray Hybridization

CD34+ HSPC cells were collected from cytapheresis from 3 patients undergoing autologous stem cell transplantation for myeloma, and purified by positive selection with magnetic beads on the Isolex 300 (Nexell Therapeutics, Irvine, CA, USA). This study was approved by the Ethical Committee of the Hôpital Saint-Eloi (CPP Sud Méditerranée IV) under the number DC-2008-417. RNA was extracted using RNeasy Kit (Qiagen, Hilden, Germany). Blood samples were collected by venipuncture from 4 healthy subjects in PAXgene® collection tubes (PreAnalytix/Qiagen, Courtaboeuf, France). All samples were collected after informed consent. PAXgene® collection tubes provide a simple mean for standardized blood collection and RNA stabilization for prolonged time at room temperatures [6]. Total RNA was extracted with PAXgene® Blood RNA kit (Qiagen, Courtaboeuf, France), including DNase I treatment. RNA purity and integrity was assessed by capillary electrophoresis using the Bioanalyzer 2100 (Agilent, Santa Clara, CA, USA) and the 2100 Expert software. All samples displayed a mean RNA Integrity Number (RIN) of at least 7.6. In order to improve microarray sensitivity, globin RNA species were removed from blood samples using the GLOBINclear® kit (Ambion, Austin, TX, USA). Two micrograms of total RNA were then used for sample labeling. Ribosomal RNA reduction, first doublestranded cDNA synthesis, cRNA synthesis, second round single-strand (ss) cDNA synthesis, ss-cDNA fragmentation, hybridization to the Human Exon 1.0 ST microarray (Affymetrix, Santa Clara, CA, USA) and scanning was processed according to the manufacturer's instructions. The microarray data are accessible at the US National Center for Biotechnology Information, Gene Expression Omnibus (GEO) (http://www.ncbi.nlm.nih.gov/geo/) with the series accession number GSE15207.

### Microarray Data Analysis

In order to compare blood and CD34 to other tissues, we used a transcriptome collection of 11 human control tissues: breast, cerebellum, heart, kidney, liver, muscle, pancreas, prostate, spleen, testis and thyroid. This dataset (3 samples per tissue) is provided by Affymetrix (http://www.affymetrix.com/support/technical/sample_data/exon_array_data.affx).

All samples were normalized before analysis with the GCOS 1.4 software (Affymetrix), (i.e. conservation of the 75^th^ percentile of each probecell). Sets of probeset (PS) have been distinguished based on the level of evidence supporting the existence of the transcripts in each set. The “core” PS set targeting RefSeq whereas the “full” PS set adds PS evidenced by ESTs or/and sequence prediction only. Expression signal values and p-values were obtained for each PS using the Robust Microarray Analysis (RMA) algorithm in ArrayAssit® software (Stratagene, La Jolla, CA, USA), on the “full” PS set (1,381,324 PS). Quality controls showed that 96–100% control PS were detected and 63.6–69.5% of all PS. A principal component analysis (PCA) was performed using ArrayAssist® software to provide a global view of how the various sample groups were related. Background PS were discarded by a “Detection Above Background” (DABG) filter when less than 3 samples had a DABG *P*-value ≤0.05. DABG filtering reduced the PS figure to 303,595 PS, corresponding to 19,871 transcripts. For the gene level analysis, summarization of the PS dataset into a unique transcripts dataset was done with ArrayAssist® software. Supervised analysis at transcript level was performed with Significance Analysis of Microarrays (SAM) method [7] (http://www-stat.stanford.edu/~tibs/SAM/) using 100 permutations, a 2-fold ratio cut-off and a FDR <5%. Gene Ontology annotation analysis was carried out using the Fatigo+ tool at the Babelomics website (http://babelomics.bioinfo.cipf.es) [8].

### Alternative Splicing Detection Algorithm

For analysis at exon level, we used the set of 303,595 PS obtained after application of the DABG filter. Identification of alternative splice variants in hematopoietic samples was done using differential splicing indexes (dsi). We applied 3 dsi:
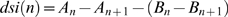


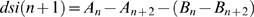


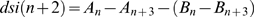



*A* and B are the mean log signal of two group of samples, respectively, that are compared in four contiguous PS numbered n, n+1, n+2 and n+3. We applied these dsi to each PS of a gene. We then computed a total dsi :


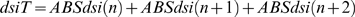
 (where ABS dsi is the absolute value of dsi) between blood or CD34+ HSPC and each one of the 10 solid tissues (spleen excluded), and between whole blood and CD34+ HSPC. We retained for each comparison the 100 PS with the highest dsiT. These 2,100 selected PS belonged to 849 different transcripts. Genome annotations and information on known transcripts were retrieved with GenBank, UCSC genome browser, X-Map and FAST DB [9], [10], [11].

### Real-Time Quantitative Reverse Transcription-PCR

Microarray results were tested by real-time quantitative reverse transcription-PCR (qRT-PCR) for 2 newly identified HSPC genes, EREG and DPPA4, and for 10 alternative splicing candidate genes: INPP4B, NEDD9, FCN2, VAV3, MBNL3, CD74, SQSTM1, PTPLA, CXCL3, COMMD6. The correspondence between PS and exon or intron numbering was done following the GenBank annotation (http://www.ncbi.nlm.nih.gov/nuccore). We used PAXgene® blood RNA, RNA from leukocytes after red blood cells lysis or from purified polymorphonuclear (CD15+), monocyte (CD14+), B cell (CD19+) or T cell (CD3+) populations isolated from a peripheral blood sample by using fuorescence-activated cell sorting (FACS) cell sorting. Purity of sorted blood cells was ≥97.8% as determined by flow cytometry (without debris). For qRT-PCR validation of DPPA4, we used as control human embryonic stem cells (hESC). HESC cell line HUES1 was imported from Douglas Melton's laboratory (Harvard University, MA, USA). The HD90/D18/FE07-142-L1 hESC cell line was derived in our laboratory from an embryo that carried an abnormal VHL gene according to preimplantation genetic diagnostic [12]. RNA was isolated from these 2 cell lines with RNeasy Mini Kit (Qiagen, Hilden, Germany). We used commercially available RNAs for CD34+ cells (StemCell technologies, Grenoble, France), breast, cerebellum, heart, liver, muscle, testis (Clinisciences, Montrouge, France). We generated cDNA from 500 ng of total RNA for each tissue using the Superscript II reverse transcriptase (Invitrogen, Cergy-Pontoise, France). Primer design for qRT-PCR was done with IDT's PrimerQuest software from Integrated DNA Technologies (http://eu.idtdna.com/Scitools/Applications/Primerquest/) according to the published sequences from GenBank and Affymetrix PS sequences of interest. Primers sequences are listed in Supplemental Table S1. PCR were carried out for 45 cycles using LightCycler® 480 SYBR Green I Master (Roche Diagnostic GmbH, Mannheim, Germany) in the LightCycler 480 instrument and normalized to *ABL* for each sample. Quantitative expression results are shown as relative expression signals compared to the less expressed tissue.

## Results

### Exome Expression Profile of Mature Blood Cells and CD34+ Stem-Progenitors Cells

To uncover specific traits of the exome expression in hematopoietic cells, we compared whole blood samples and CD34+ purified hematopoietic stem-progenitor cells (HSPC) to a compendium of 33 samples from 11 solid tissues. Whole blood samples were obtained from four healthy adults. No cell separation step was used prior to RNA extraction, hence, these samples comprised the entire range of blood cells: polymorphonuclear leukocytes, lymphocytes, monocytes, platelets and red blood cells. CD34+ HSPC were purified using a magnetic bead isolation step from patients undergoing autologous stem cell transplantation. Samples were then labeled and hybridized to GeneChip® Human Exon 1.0 ST microarrays (Affymetrix) that includes 5,362,207 different oligonucleotides, corresponding to more than 1,400,000 probesets (PS). The compendium of solid tissues was a collection of Human Exon 1.0 ST microarray data from 11 triplicate samples of normal solid tissues: breast, cerebellum, heart, kidney, liver, muscle, pancreas, prostate, spleen, testis and thyroid gland.

**Figure 1:**
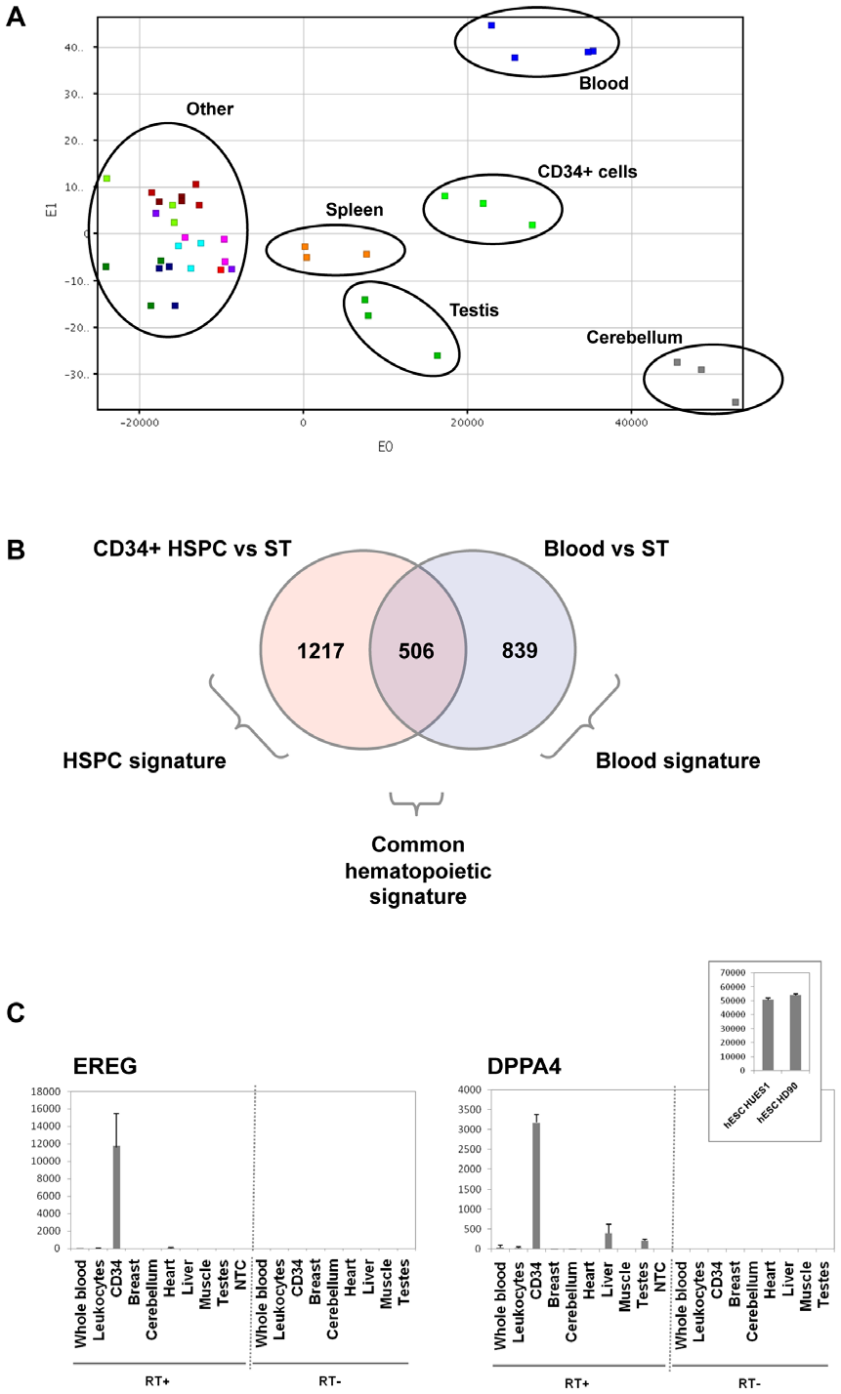
Hematopoietic exome expression analysis at transcript-level. (A) Global view of gene expression by principal component analysis (PCA) performed on 33 solid tissues samples, 3 mobilized CD34+ hematopoietic stem-progenitor cells (HSPC) and 4 peripheral whole blood samples, on the full probe sets (PS) dataset on Affymetrix Human Exon 1.0 ST microarrays. Blood cells and CD34+ HSPC displayed a distinct spatial localization, in agreement with the very specific expression profile of these cells. Of note, testis and cerebellum could also be individualized based on their global gene expression, whereas all other solid tissues clustered together. Spleen samples localized in an intermediate location, between hematopoietic and solid tissues samples, in agreement with their mixed content of both hematopoietic and non-hematopoietic cells. (B) Venn diagram detailing shared and distinct gene expression among blood cells and HSPC. The “common hematopoietic signature”, was defined as the intersection of the CD34+ HSPC signature (genes overexpressed in CD34+ HSPC compared to solid tissues (ST)) and the whole blood signature (genes overexpressed in blood cells compared to ST). (C) Real-time quantitative RT-PCR (qRT-PCR) validation of overexpression of EREG and DPPA4 genes in HSPC. QRT-PCR were performed on several solid tissues, on whole blood and purified leukocytes and on CD34+ samples (RT+). Results showed specific expression of EREG (left) in CD34+ cells and expression of DPPA4 (right) in HSPC and in 2 human embryonic stem cell (hESC) cell lines, HUES1 and HD90. No Template Control (NTC): qRT-PCR without any nucleic acid sample. RT- : qRT-PCR control without reverse transcriptase, demonstrating the absence of DNA contamination. Results are shown as relative expression signals compared to the less expressive tissue (signal at 1). All qRT-PCR were performed twice.

A principal components analysis (PCA) on all samples showed that blood cells and HSPC segregated in a different spatial localization from all other solid tissues tested, substantiating the very specific expression profile of these cells (Figure 1A). We found that testis and cerebellum also displayed a specific signature, different from other tissues, as previously reported by others [13]. Spleen samples localized in an intermediate location, between hematopoietic and solid tissues samples, in agreement with their mixed content of both hematopoietic and non-hematopoietic cells. Spleen samples were therefore excluded from the analysis, to prevent these samples with ambivalent composition to confuse the analysis based on a comparison between hematopoietic and non-hematopoietic groups.

### Gene Level Analysis Highlights Hematopoietic Specific Gene Expression Signatures

A first global analysis was carried out at gene level, summarizing all PS signal values from all exons from a given gene into one unique value. Using this approach, the full PS dataset was summarized into a 19,871 unique transcripts dataset. Using a significance analysis of microarrays (SAM) with a 2-fold ratio cut off and a false discovery rate (FDR) <5%, we compared whole blood and CD34+ HSPC to the group of solid tissues samples. 1345 and 1723 transcripts were found up regulated in blood and CD34+ HSPC samples, respectively. By intersecting these lists of genes (see Venn diagram in Figure 1B), we observed that 506 genes were up regulated in both blood and CD34+ HSPC, composing a “common hematopoietic signature”, whereas 839 transcripts were specifically up regulated in blood cells (“whole blood signature”) and 1217 in CD34+ HSPC cells (“HSPC signature”) (Supplemental Table S2). The common hematopoietic signature comprised genes involved in cell movement (chemotaxis, homing, rolling, infiltration, extravasation and transmigration) including ITGB2, ITGA4, ITGAL, SELL, CXCR4 and CD44. The whole blood signature comprised genes specific of each major blood cell sub-population: granulocytes (IL8RB, NCF1 and ADAM8), monocytes (LILRA1, CCR2, CD1D), B lymphocytes (CD79A, CD180), T lymphocytes (IL7R, CD3G, CD27), platelets (CCL5, PF4 and NRGN) and reticulocytes (ALAS2, HBA2, HBA1). These later transcripts correspond to the mRNA present in reticulocytes and are remnant of the erythroid progenitors transcriptome [14]. The whole blood signature was also significantly enriched in biological pathways involved in calcium metabolism and tyrosine phosphorylation and cell surface receptor linked to signal transduction. The HSPC signature comprised of note the CD34 antigen, contained known HSPC genes such as prominin (CD133/PROM1), but also genes that were not previously linked to HSPC such as epiregulin (EREG) or Developmental pluripotency-associated gene 4 (DPPA4). QRT-PCR validation showed a strong overexpression of EREG and DPPA4 in CD34+ cells (figure 1C), confirming microarray analysis. EREG was 70-fold more expressed in HSPC than in other tissues. We also validated expression of DPPA4 in two human embryonic stem cell (hESC) cell lines, HUES1 and one of our laboratory derived cell line, HD90. We identified genes that were underexpressed in whole blood and HSPC cells compared to solid tissues (Supplemental Table S2). The most prominent findings were the loss of genes involved in tissue cohesion such as tight junctions (claudins CLDN5 and CLDN8, the tight junction protein TJP1), gap junctions (GJA1, GJA7) and anchoring junctions (cadherins, integrins, notably ITGA1, desmoplakin, plakophilins and other focal adhesion components such as talin, focal adhesion kinase like protein tyrosine kinase 2 (PTK2)). Of note, genes involved in various metabolism pathways, in particular amino acids metabolism and urea cycle were significantly underexpressed in blood cells, illustrating some fundamental differences in metabolism between circulating blood cells and solid tissues. Altogether, these results validate the biological relevant of our data and point to previously unrecognized HSPC markers.

### Exome Expression Data Visualization Using the Amazonia Exon! Database

**Figure 2:**
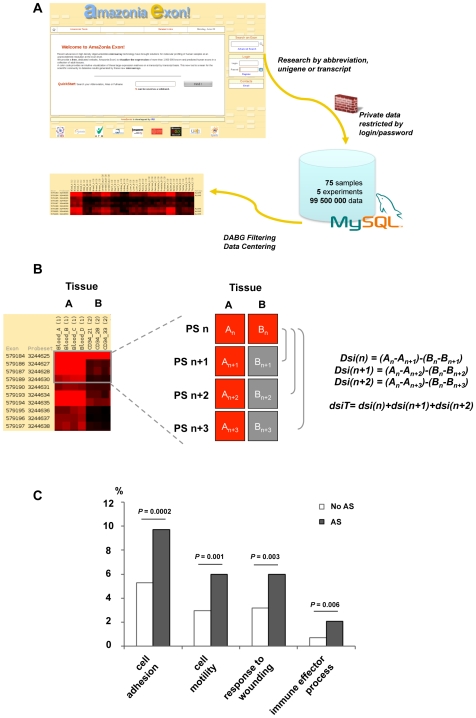
Alternative splicing discovery with exon array. (A) Exon expression profiles on GeneChip® Human Exon 1.0 ST microarray can be viewed as colored matrices on the Amazonia Exon! web tool. Transcripts are accessed by key words and are visualized as matrices with samples in columns and PS in lines. The exon and PS ID according to Affymetrix numbering are provided. A color code provides the relative or absolute expression level of each exon in each sample. DABG filtering can be performed to exclude background PS. Blood samples and CD34+ HSPC samples are compared to 11 solid tissues. (B) Alternative splicing detection algorithm: pattern searched, corresponding to a differential expression between 2 different tissues (left). Three differential splicing indexes (*dsi*) were applied. *A* and *B* are the mean log signal in tissues A and B in four contiguous PS numbered n, n+1, n+2 and n+3 (right). These dsi were applied to each PS of a gene. A total *dsiT* for each PS was then computed. (C) Biological function of AS genes in hematopoietic cells. The 849 transcripts of the “hematopoietic AS” list were significantly enriched in genes involved in immune effector processes (*P* = 0.006), response to wounding (*P* = 0.003), cell motility (*P* = 0.001) and cell adhesion (*P* = 0.0002) The statistical analysis was carried out using the Babelomics webtool (http://babelomics.bioinfo.cipf.es/). Gene Ontology “biological process” categories which differed significantly (*P-*value≤0.01) between non AS genes (bright bars) and AS genes in hematopoietic cells (dark bars) are shown.

A dedicated website was constructed, Amazonia Exon! (http://amazonia.transcriptome.eu/exon.php), to display the ∼50 millions data points analyzed in this study as heatmaps on a gene per gene basis (Figure 2A). Transcripts are accessed by key words and are visualized as a colored matrix with samples in columns and PS in lines. A filtering tool is available to exclude from the graphic representation PS whose expression remains within background for most samples. A link in the Amazonia Exon database is inserted under each colored matrix. This link leads to Affymetrix web site, NetAffx™, where the user can obtain all information about the transcript cluster and the different PS. This tool was used all along our analysis to instantly visualize this complex dataset. Hence, the scientific community has free access to this atlas for a graphical representation of the exome expression landscape in whole blood, CD34+ HSPC and 11 different adult tissues.

### Identification of Differentially Regulated Splice Variants between Hematopoietic Cells and Solid Tissues: Preferential Involvement of Cell Motility and Immune Response Genes

Having defined the entire expression map of the human exome in hematopoietic cells, we examined the exons that would be involved in alternative splicing events. To this end, we devised a differential splicing index (*dsiT*) based on the differential expression of a short series of PS between two different tissues (see material and method and Figure 2B). The development of *dsiT* was based on the reasoning that a differential splicing index is more robust to variation of the dynamic range of PS from a same gene than a splicing index comparing the expression level of one PS to a global gene value. Using this index, we compared whole blood and CD34+ HSPC samples to each solid tissue and obtained a “hematopoietic AS” list composed of 849 different transcripts (Supplemental Table S3). Among these transcripts, some are known to undergo AS events in hematopoietic tissues, such as EZH2 [4], or in other tissues such APP [15] and TPM1 [16]. The hematopoietic AS list contained transcripts clearly overexpressed in mature whole blood cells or CD34+HSPC such as CD74, GALNAC4S-6ST, ZNRF1 or RAB37 while other are not, such as NEDD9, AKR1C1, TRIM58 or APOD. Among the 2562 genes up-regulated in HSPC and/or blood cells, 108 are comprised in the “hematopoietic AS” list. Among the 4018 down-regulated genes, 276 genes are alternatively spliced. Representative examples of differential splicing event between mature blood cells or HSPC and solid tissues are shown in Supplemental Figure S1. Strikingly, we observed that the functional annotations of these 849 transcripts were significantly enriched in genes involved in immune effector processes (*P* = 0.006), response to wounding (*P* = 0.003), cell motility (*P* = 0.001) and cell adhesion (*P* = 0.0002) (Figure 2C). As these processes are central to the specific biological properties of hematopoietic cells, our results illustrate that AS indeed mediate important aspects of blood cell differentiation and function. The 50 first hematopoietic AS transcripts are listed in Table 1.

**Table 1:** Fifty representative transcripts undergoing AS between whole blood cells or HSPC and diverse solid tissues from the complete “hematopoietic AS” list.

Probe Set ID	Transcript Cluster ID	Gene symbol	Link to Amazonia Exon!
2941833	2941784	NEDD9	2941784
2390191	2390180	TRIM58	2390180
3233013	3232979	AKR1C1	3232979
3668877	3668834	ZNRF1	3668834
3734435	3734413	RAB37	3734413
3850270	3850261		3850261
2508634	2508611	ARHGAP15	2508611
2773395	2773387	CXCL3	2773387
3125258	3125116	DLC1	3125116
3280005	3279982	PTPLA	3279982
2714685	2714672	MAEA	2714672
2649937	2649824		2649824
2787556	2787459	INPP4B	2787459
3008175	3008164	LAT2	3008164
2844529	2844479	SQSTM1	2844479
2642361	2642325		2642325
2881401	2881370	CD74	2881370
2477160	2477073	CRIM1	2477073
3355734	3355733		3355733
3518177	3518169	COMMD6	3518169
3200662	3200648	ADFP	3200648
2495898	2495881		2495881
3597355	3597338	TPM1	3597338
3024031	3024025	MEST	3024025
3113283	3113280		3113280
3283123	3282974	SVIL	3282974
3625604	3625539	NEDD4	3625539
3775851	3775842		3775842
2835602	2835576	SYNPO	2835576
2487210	2487082	ANTXR1	2487082
2650544	2650538	NMD3	2650538
2736328	2736322	PDLIM5	2736322
3268942	3268940	GALNAC4S-6ST	3268940
2440958	2440943		2440943
3110230	3110217	BAALC	3110217
3438109	3438061		3438061
3577642	3577612	SERPINA1	3577612
2840038	2840110	DOCK2	2840110
3948673	3948640	FBLN1	3948640
3952765	3952762	CLDN5	3952762
2586369	2586348	METTL5	2586348
3663324	3663287	NDRG4	3663287
3852919	3852880		3852880
3871112	3871095	TNNT1	3871095
2603466	2603460	NCL	2603460
3244635	3244622		3244622
2902430	2902427	LST1	2902427
4054214	4054204	APOD	4054204
3822666	3822657		3822657
3560684	3560673	CFL2	3560673

**Figure 3:**
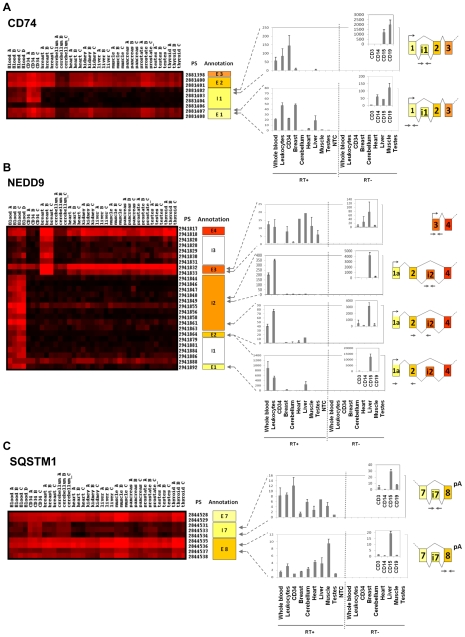
Differential exon expression between hematopoietic cells and solid tissues. qRT-PCR validation for 3 transcripts differentially expressed between hematopoietic cells and solid tissues: CD74 (A), NEDD9 (B) and SQSTM1 (C). Each transcript is defined with its gene name and transcript number and can be visualized as a colored matrix in Amazonia Exon! web site. Expression level is color coded from no expression (black) to high-level expression (red). The exon and PS ID according to Affymetrix numbering are provided on the right of each matrix. Global gene expression can be visualized on http://amazonia.transcriptome.eu/exon.php. For each transcript, a zoom on the expression matrix obtained in Amazonia Exon! is displayed with PS ID. The correspondence between PS and exon/intron numbering according to GenBank is shown on the right of the cluster (E: exon, I: intron, NA: Not Annotated). Arrows represent PCR primers positions. Experimental validation of alternative splicing events by qRT-PCR in whole blood, leukocytes, CD34+ HSPC cells, breast, cerebellum, heart, liver, muscle, testes and CD3+, CD14+, CD15+, CD19+ positive purified populations (RT+). No Template Control (NTC): qRT-PCR without any nucleic acid sample. RT- : qRT-PCR control without reverse transcriptase, demonstrating the absence of DNA contamination. Results are shown as relative expression signals compared to the less expressive tissue (signal at 1). All qRT-PCR were performed at least twice. (A) Our splicing index discovery scheme led us to detect expression of an intronic sequence in CD74 gene specifically in blood and HSPC. Experimental validation confort this result, showing a part of intron 1 expression in CD15 and CD19+ cells. (B) Exon microarray data showed a long form of NEDD9 specifically expressed in blood cells, whereas a short isoform was detected in all tissues studied. QRT-PCR confirmed that a long form was specifically expressed in blood cells, notably in CD15+ cells. Expression of long parts of intron 2 of NEDD9 gene detected by exon array was also validated by qRT-PCR, suggesting a novel exon due to intron retention. (C) Several PS located in intron 7 of the SQSTM1 gene were expressed according to microarray data. We validated the retention of intron 7 by qRT-PCR, showing an overexpression in CD34 cells.

**Figure 4:**
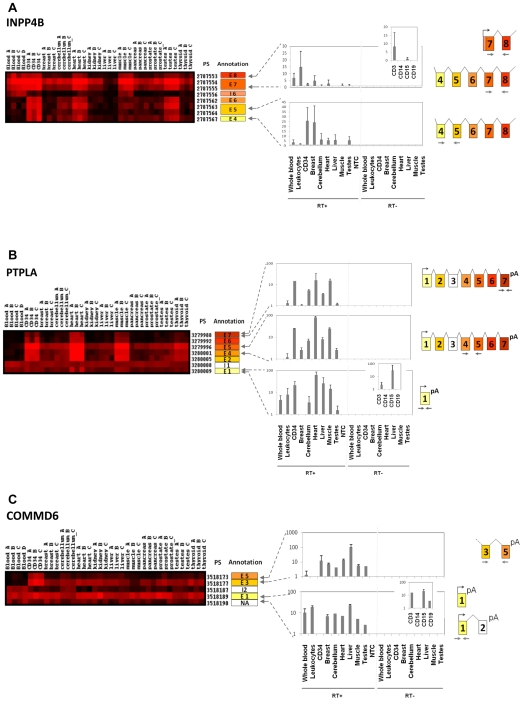
Differential exon expression between whole blood and HSPC: splice isoform switch during hematopoietic differentiation. Expression matrices and experimental validation of microarray results for INPP4B, PTPLA and COMMD6 (see legend for Figure 3). (A) Exon microarray results for INPP4B gene showed that exons 4, 5, and 6 were under expressed in blood samples compared to CD34+ samples. Interestingly, a PS situated in intron 6 showed an expression similar to that of exon 7, suggesting an exon extension in exon 7 in blood cells and existence of an alternative promoter just upstream of this exon. QRT-PCR results confirmed detection of exons 7 and 8 in whole blood and in leukocytes, notably in CD3+ T-cells, whereas exons 4 and 5 were not expressed in blood samples but detected in CD34 cells. These results point out the absence of a long transcript (including exons 4 and 5) in mature blood cells. (B) The exome expression profile showed that blood express only a short transcript form of PTPLA, limited to exon 1, whereas CD34+ HSPC cells and some solid tissues expressed a long form spanning exon 1 to exon 7. We showed by qRT-PCR that blood and specifically CD3+ and CD15+ cells expressed the short form corresponding to exon1, but not the long form including exon 7. (C) Hematopoietic cells show differential expression of COMMD6 gene: microarray data indicate expression of one unique exon (exon 1) in blood, whereas CD34+ HSPC and other solid tissues expressed transcripts containing exons 3 to 5. This differential expression was confirmed by qRT-PCR, and suggested at least two alternative splicing variants for COMMD6 with a mutually exclusive expression mode during hematopoietic differentiation.

Ten transcripts, involved in cell motility or immune response, were selected for validation by qRT-PCR: NEDD9, MBNL3, VAV3, INPP4B, FCN2, CD74, COMMD6, PTPLA, CXCL3 and SQSTM1. We confirmed the microarray results for 6 transcripts (60%): INPP4B, CD74, PTPLA, NEDD9, COMMD6 and SQSTM1. These genes showed clear alternative splicing expression patterns, including potential new initiation or termination sites, as well as intron retention (Figures 3 and 4). INPP4B, PTPLA and COMMD6 showed, in addition to splicing differences between blood cells and solid tissues, a switch in alternative splicing during blood differentiation (Figure 4) and are therefore described in more detail in a specific paragraph.

The differential splicing index analysis detected that four successive PS of the gene coding for the cell surface antigen CD74, corresponding to its first intron, were specifically expressed in blood and CD34+ HSPC (Figure 3A). Our experimental validation confirmed this observation, and excluded the hypothesis of DNA contamination of our samples (Figure 3A). QRT-PCR on purified leucocytes sub-populations showed that this intron was specifically retained in B lymphocytes and CD15+ sorted granular cells. CD74 molecule, a major histocompatibility complex class II-associated invariant chain, is a known regulator of antigen processing and also plays a role in cell motility [17]. Intronic sequence expression between exon 1 and exon 2 may impact on the function of CD74, because it is located within the trimerisation domain of the protein. Neural precursor cell-expressed, developmentally downregulated gene 9 (NEDD9/CAS-L/HEF1), encodes for an adaptater protein involved in the regulation of cell division, cell proliferation and cell movement [18]. Exon microarray data showed a long form specifically expressed in whole blood cells whereas a short isoform was detected in all solid tissues studied and confirmed by qRT-PCR (Figure 3B). Here we report for the first time that the long form is specifically expressed in blood cells, notably in CD15+ cells. There was no expression of NEDD9 in CD34+ HSPC. Moreover, exon arrays showed the expression of long parts of intron 2 of NEDD9 gene. This intron retention in blood samples was also confirmed by qRT-PCR. Finally, several PS located in intron 7 of the sequestosome 1 (SQSTM1) gene were expressed according to the microarray data (Figure 3C). QRT-PCR experiments confirmed the exon data showing this intron retention notably in CD34 cells.

### Splice Isoform Switch during Hematopoietic Differentiation

We then focused on exons differentially expressed between CD34+ HSPC and mature blood cells, to explore alternative splicing events during hematopoietic differentiation. Many transcripts showed AS motif between mature and immature cells and some illustrative examples are displayed in Supplemental Figure S2. Three of the alternative splicing events involving a switch in the exon expression profile during hematopoietic stem cells differentiation into mature hematopoietic cells were further validated by qRT-PCR: INPP4B, PTPLA and COMMD6. Inositol polyphosphate 4-phosphatase type II (INPP4B) encodes a phosphatase involved in the regulation of phosphatidylinositol-3-kinase (PI3K) mediated signal transduction. Exome expression profile of INPP4B showed that exons 4, 5, and 6 were under expressed in blood samples compared to CD34+ HSPC and solid tissues (Figure 4A). In addition, a PS situated in intron 6 displayed an expression profile similar to that of exon 7, suggesting an exon extension in exon 7 in whole blood cells. As neither exons 1, 2 or 3 were detected in whole blood cells (data not shown), these results suggest strongly the existence of an alternative promoter just upstream of the extended exon 7 that is used in blood cells. Figure 4A shows the qRT-PCR results confirming the absence of exon 4 and 5 expression in whole blood, whereas expression of exon 7 and 8 is evidenced, notably in CD3+ T-cells, in addition to CD34 cells, breast, cerebellum, heart, liver and testes samples. These results were concordant with the microarray results and demonstrated the absence of the long transcript in mature blood cells. Protein tyrosine phosphatase-like a (PTPLA) is a protein tyrosine phosphatase-like that has the highly conserved arginine residue of the conventional tyrosine phosphatase domain replaced by a proline residue [19]. The exome expression profile and our experimental validation showed that blood (specifically CD3+ and CD15+ cells) express only a short transcript form, limited to exon 1, whereas CD34+ HSPC cells and solid tissues expressed a long form spanning exon 1 to exon 7 (Figure 4B). EST databases confirmed the existence of these two alternative splicing forms, but our results specifically show that this gene switches from a short to a long form during hematopoietic differentiation. Finally, we looked at the expression of COMMD6 which belongs to a family of NF-kappa-B-inhibiting proteins that contain a hypertension-related, calcium-regulated gene HCaRG domain (HCaRG) involved in the control of cell proliferation [20]. This gene was expressed in whole blood cells as a short transcript form containing only exon 1, whereas CD34+ HSPC expressed transcripts containing exons 3 to 5 (Figure 4C). This differential expression was confirmed by qRT-PCR, and suggested that this gene code for at least two alternative splicing variants with a mutually exclusive expression mode during hematopoietic differentiation.

## Discussion

While it is acknowledged that AS contributes extensively to transcript and protein complexity and sophistication, it is still not often taken into consideration in functional studies of hematopoietic cells. This was mainly due to the lack of tools to apprehend AS at the exome level. We took advantage of the Affymetrix Exon ST 1.0 microarray, which measure the expression level of more than one million different known or predicted human exons, to uncover blood and CD34+ HSPC AS. This dataset was compared to a collection of 11 solid tissues, in a search of AS that would be hematopoietic specific and possibly underlying hematopoietic functions such as cell motility and immune response.

A first comparison was carried out at a whole transcript level and confirmed the microarray data validity with a strong expression of either mature blood cell type genes in whole blood or stem cell markers in HSPC. Interestingly, HSPC also markedly expressed EREG and DPPA4, as confirmed by qRT-PCR. Epiregulin (EREG) is a member of the epidermal growth factor family and is used as a marker of several cancers [21], [22]. It binds to ERBB1/EGFR and ERBB4 receptors. Microarray data show low ERBB1, and non ERBB4 expression in HSPC, thus leaving open the possible autocrine role of EREG in these cells. We showed that DPPA4 was expressed in human pluripotent cells, as others [23], and could be related to the stemness state of HSPC. The expression of these two genes in HSPC was not previously reported to the best of our knowledge. We also confirmed this expression pattern on independent microarray data (data not shown but can be accessed on our microarray expression atlas Amazonia! (http://amazonia.transcriptome.eu/)).

However, the strength of the exon arrays is to provide an unbiased profile of the human exome expression, including novel variation in splicing [24]. Indeed, a large set of differential splicing event was evidenced, including alternative splicing, alternative donor or acceptor site, intron retention, and alternative first or last exons (Figures 3 and 4 and Supplemental Figures S1 and S2). For the first time, a large number of differential exon usage is listed in undifferentiated and mature hematopoietic cells (Table 1 and supplemental Table S3). We validated by qRT-PCR 60% (6/10) of the transcripts selected for validation (CD74, NEDD9, SQSTM1, INPP4B, PTPLA, COMMD6). This conservative percentage is similar to what is found by other groups analyzing data provided by the Whole Exon ST 1.0 microarray [25], [26]. Remarkably, alternatively spliced genes were significantly enriched in genes playing a role in cell motility, cell adhesion, response to wounding and immune processes. As these functional annotations are attributes of hematopoietic cells, these results compellingly suggest that splicing diversity is involved in the molecular mechanisms that mediate hematopoietic function and differentiation. AS events were detected between immature and mature cells suggesting a role of AS during differentiation. For instance, genes such as PTPLA, or the RHO GTPase ARHGAP15 undergo a change in exon composition during hematopoietic cell maturation. We asked whether these splicing events could target known functional domains. A search in the PFAM database revealed that the first intron of CD74, which is retained in mature and immature hematopoietic cells, disrupt the trimerisation domain and is thus expected to modify the functionality of this gene that is also involved in cell movement. However, the other validated splicing occurrences did not affect protein domains listed in public databases (data not shown), implying that the splicing instead impacted on domains that are specific of these genes. For PTPLA and COMMD6, we detected 5′ short transcripts specifically expressed in blood cells. These short transcripts could interfere with gene expression regulation [27], [28].

The inventory of splicing events occurring in human cells had until recently been hampered by technical biases such as a 3′ bias and insufficient coverage for expressed sequence tag (EST)-derived cDNAs or bias toward a predetermined and limited set of alternative splicing events for junction arrays [1]. These limitations contrast to the extensive and unbiased coverage of exon arrays that probe for the expression of most known or predicted exons, including sequences tagged as introns but that could be in fact exons. However, though we detected many alternative splicing events using the whole exon chip, we are aware that these microarrays are not be able to detect exhaustively all alternative splicing in a given tissue. Indeed, these microarrays measure individually the expression of each exon and can therefore not appreciate whether the signal detected is the result of the expression of one unique type of transcript or the mix of different splicing products. Deconvolution techniques have been proposed to extrapolate from the Exon ST 1.0 data the various transcripts present in the sample, but the complexity of the task may be beyond current computational resources. The next step will be mRNA sequencing, a technique still in its infancy and whose sensitivity will be challenged by the high prevalence of alternatively spliced variant occurring at low copy number and that may be to the consequence of splicing noise [29]. In this respect exon array identify only predominant splice variations, more likely playing strong functional roles.

In conclusion, this work establishes at an unprecedented high resolution the expression profile of the human exome, and hence an extensive panorama of splicing, in hematopoietic cells. By making these data publicly available through our website Atlas (Amazonia Exon!), this reference work is made easily accessible to the medical and scientific community, which is a sine qua non condition to promote the study of alternative splicing in hematology.

## Supporting Information

Figure S1 Examples of differential exon expression between hematopoietic cells and solid tissues. Exon array results are visualized with Amazonia Exon! Candidate genes selected with our AS detection algorithm showed clear differential PS expression between whole blood samples (A, B, C, D) or HSPC samples (A, B, C) and solid tissue samples (A, B, C). The exon and PS ID according to Affymetrix numbering are provided on the right of each matrix as well as the GenBank sequence correspondence. Global gene expression can be visualized on http://amazonia.transcriptome.eu/exon.php.
(0.65 MB TIF)

Figure S2 Examples of differential exon expression between whole blood and HSPC. The alternative splicing discovery tool identified several transcripts differentially expressed between mature blood cells and CD34+ cells (see legend for Supplemental Figure S1).
(0.72 MB TIF)

Table S1 Primers sequences for qRT-PCR validation.
(0.03 MB XLS)

Table S2 Gene-level analysis: Blood cells and HSPC signatures. 839 genes were specifically upregulated in blood cells, 1217 in CD34+ cells, and 506 in both blood and HSPC. 2187 genes were specifically down-regulated in blood cells, 390 in HSPC and 1441 in common.
(0.79 MB XLS)

Table S3 Exon-level analysis. 849 transcripts undergoing splicing between hematopoietic cells and solid tissues (“hematopoietic AS”), ordered by global splicing pattern.
(0.18 MB XLS)
