# Supplementary figures and images for: Expression Map of the Human Exome in CD34+ Cells and Blood Cells: Increased Alternative Splicing in Cell Motility and Immune Response Genes
Source: PLoS One. 2010 Feb 1;5(2):e8990. doi: 10.1371/journal.pone.0008990 (PMC2813875; doi:10.1371/journal.pone.0008990)

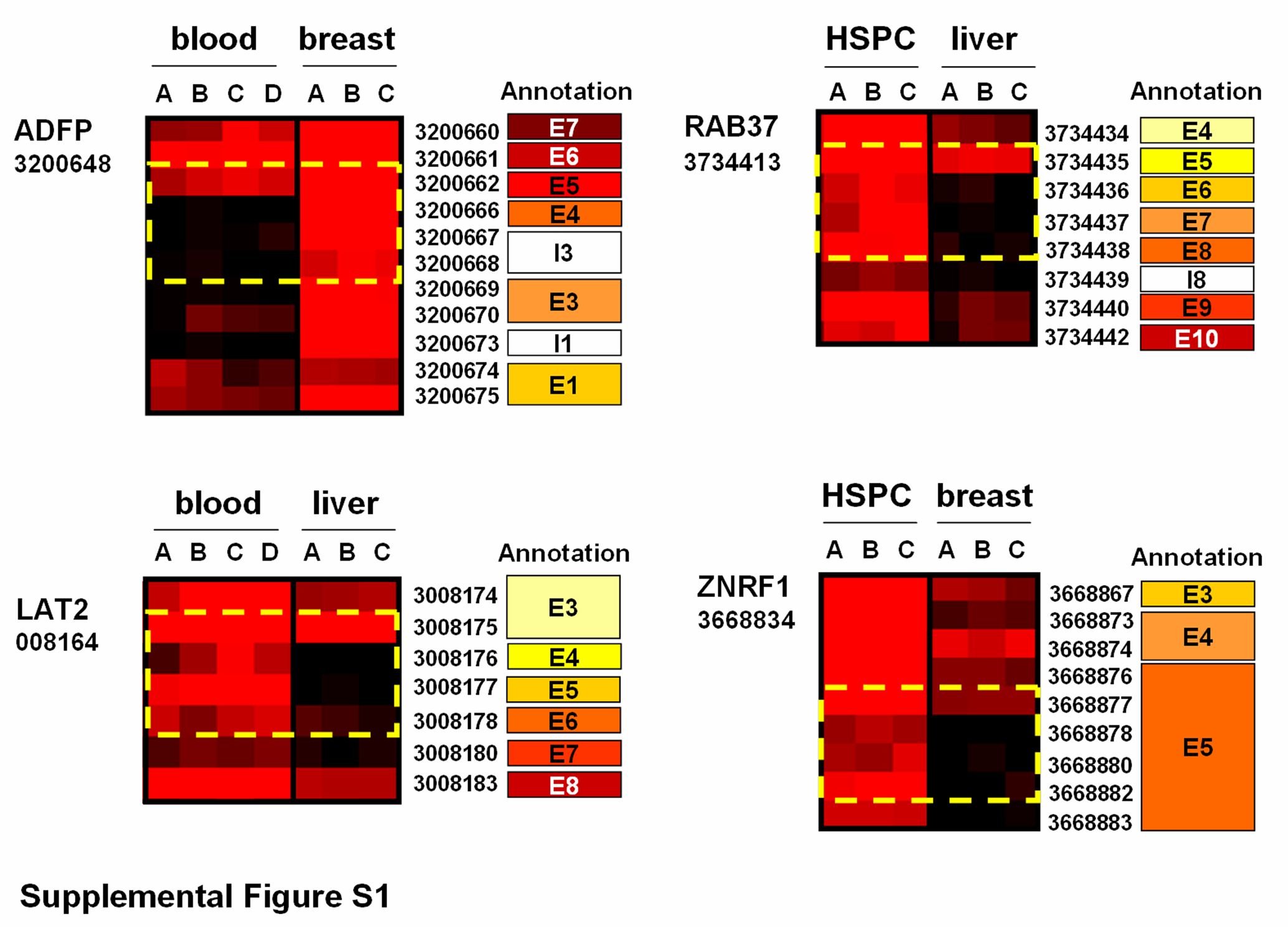

Supplement: Figure S1 — Examples of differential exon expression between hematopoietic cells and solid tissues. Exon array results are visualized with Amazonia Exon! Candidate genes selected with our AS detection algorithm showed clear differential PS expression between whole blood samples (A, B, C, D) or HSPC samples (A, B, C) and solid tissue samples (A, B, C). The exon and PS ID according to Affymetrix numbering are provided on the right of each matrix as well as the GenBank sequence correspondence. Global gene expression can be visualized on http://amazonia.transcriptome.eu/exon.php. (0.65 MB TIF) [file pone.0008990.s001.tif]

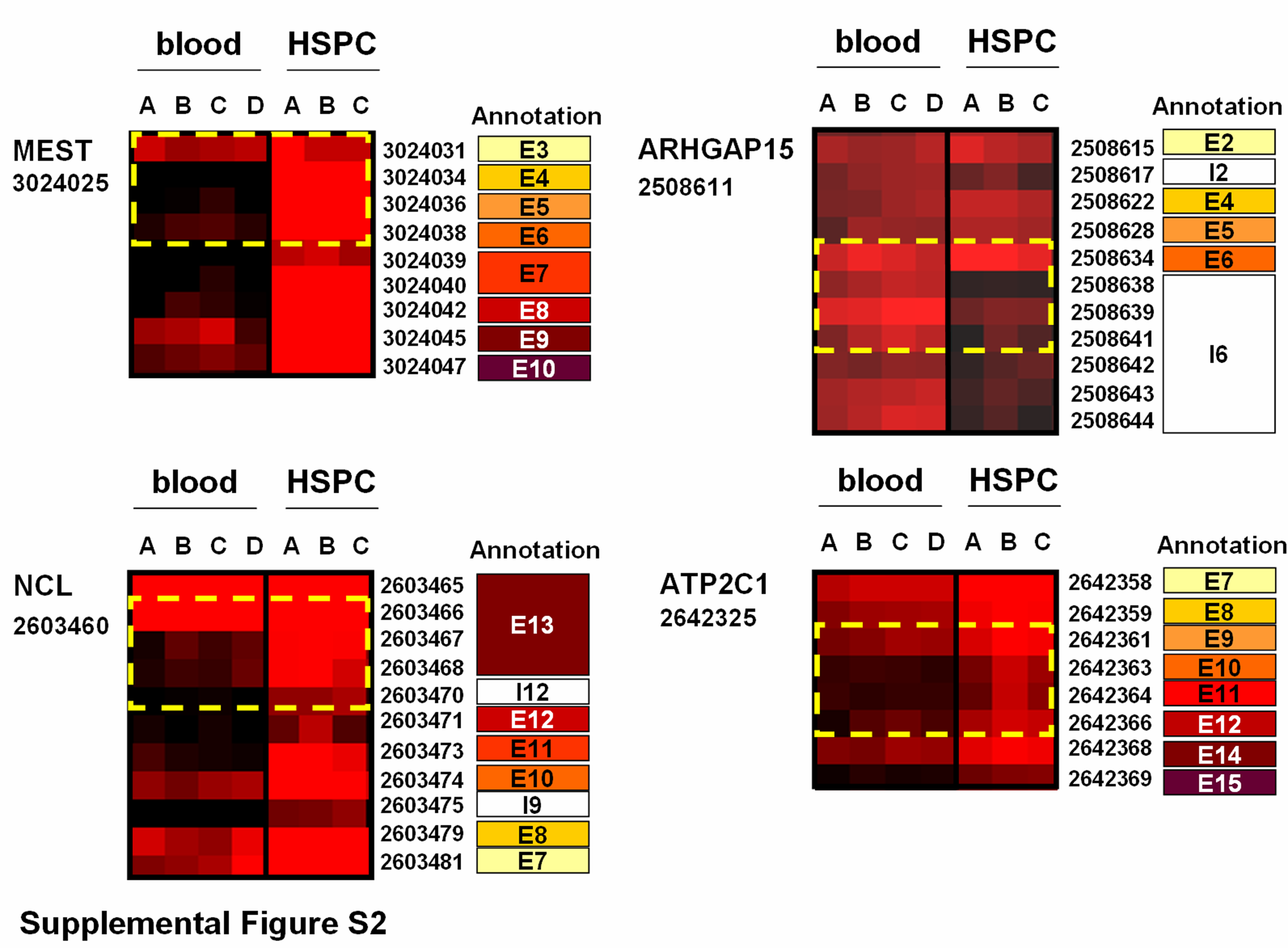

Supplement: Figure S2 — Examples of differential exon expression between whole blood and HSPC. The alternative splicing discovery tool identified several transcripts differentially expressed between mature blood cells and CD34+ cells (see legend for Supplemental Figure S1). (0.72 MB TIF) [file pone.0008990.s002.tif]
